# Supplementary material for: Simultaneous multi-patch-clamp and extracellular-array recordings: Single neuron reflects network activity
Source: Sci Rep. 2016 Nov 8;6:36228. doi: 10.1038/srep36228 (PMC5099952; doi:10.1038/srep36228)
Supplement: Supplementary Information [file srep36228-s1.pdf]

Supplementary

Simultaneous multi-patch-clamp and extracellular-array recordings: Single neuron reflects network activity

Roni Vardi†, Amir Goldental†,Shira Sardi, Anton Sheinin and Ido Kanter*

†These authors contributed equally to this work.

*Correspondence: ido.kanter@biu.ac.il

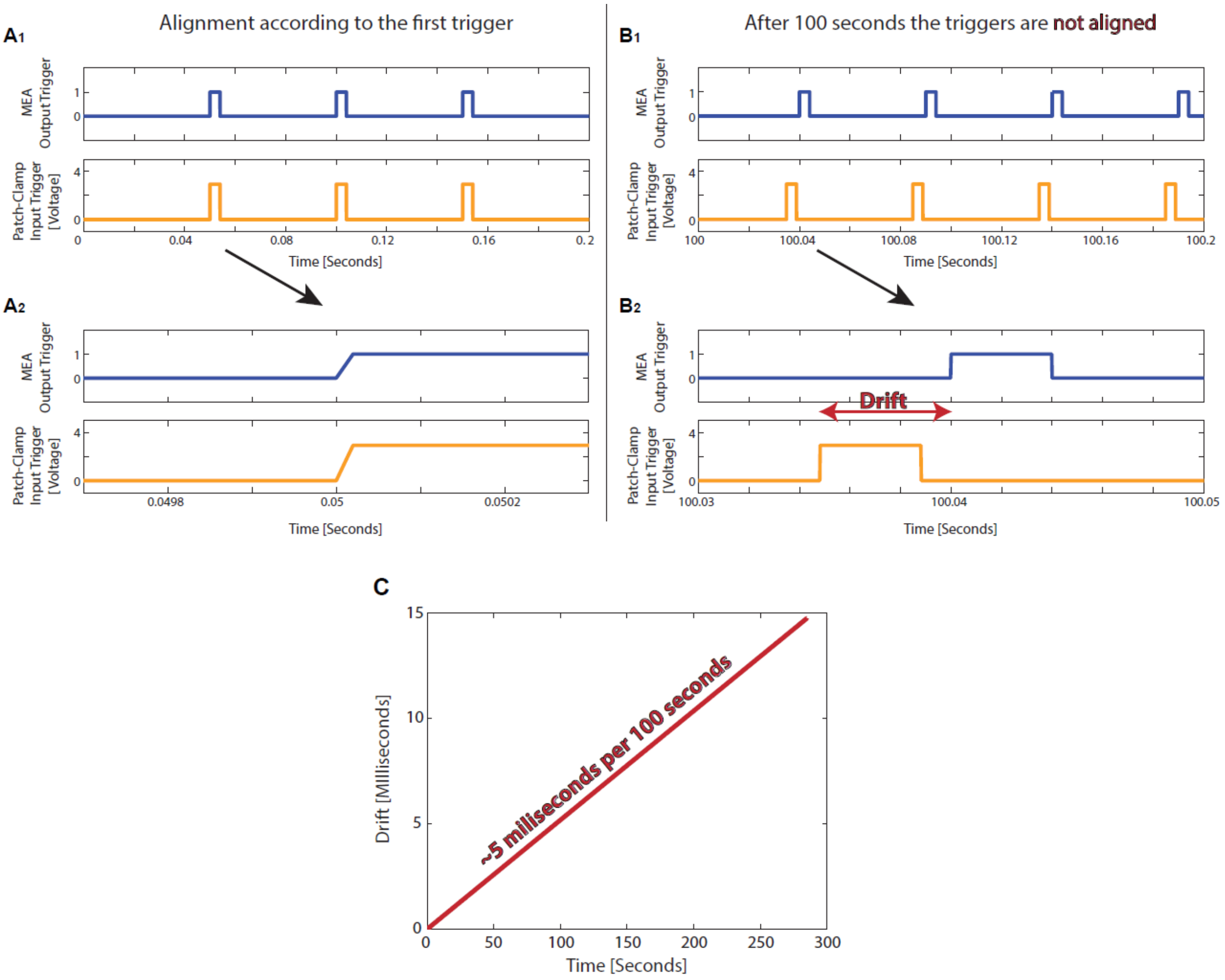

Figure S1. The drift between the two clocks. The synchronization between the two clocks is implemented using triggers, sent in a leader-laggard configuration (Figure 1 in the manuscript) from the MEA to the patch subsystem (blue curves in panels A and B). The output trigger is sent every 50 milliseconds from the MEA and its duration is 4 ms (however only the rise timing of the pulse is important and has a duration of 20 microseconds). The recorded input trigger to the patch subsystem is 3.8 V and its duration is 4 ms too (orange curves in panels A and B). Initially, the recoded data by the MEA and patch clamp systems are aligned through the first trigger (panels A_1 and A_2). The drift between the clocks after ~100 seconds is visible (panels B_1 and B_2) and is estimated quantitatively (panel C). This drift was carefully balanced and canceled out in the presented data.

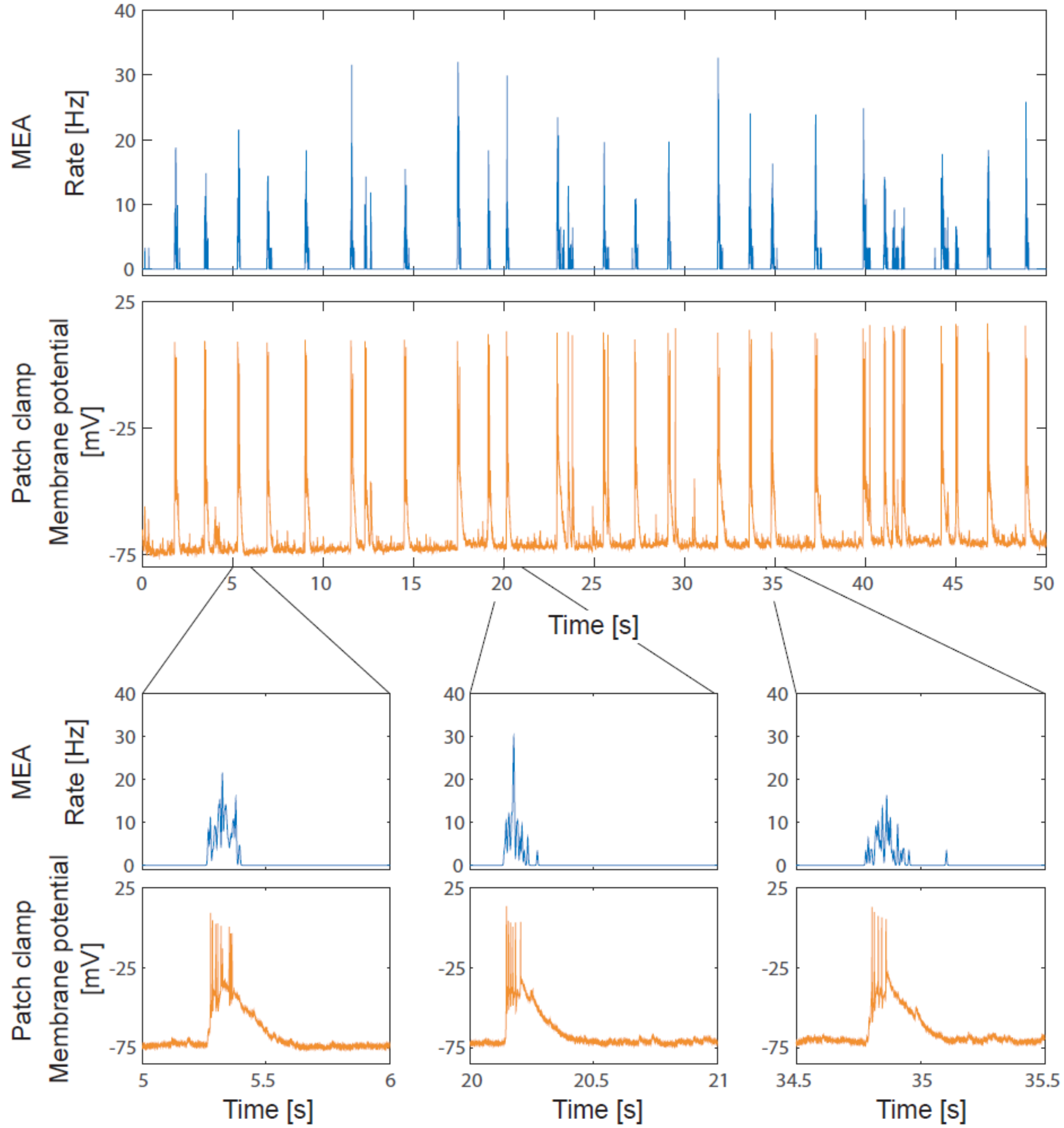

Figure S2. Another example of 50 seconds recording. Similar to Figure 2 in the manuscript, indicating that a single neuron current-clamp recording reflects the cooperative network burst activities recorded by an extracellular multi-electrode array (MEA). Upper panel: The temporal rate activity of the multi-electrode array. Middle panel: The membrane potential of the current-clamped neuron indicating high correlation with the MEA recordings. Lower panels: Zoom-in of three of the bursts shown in the upper panels. Results indicate high correlation between the bursts of the network and the single neuron internal dynamics, with a short time shift of less than 20 milliseconds.

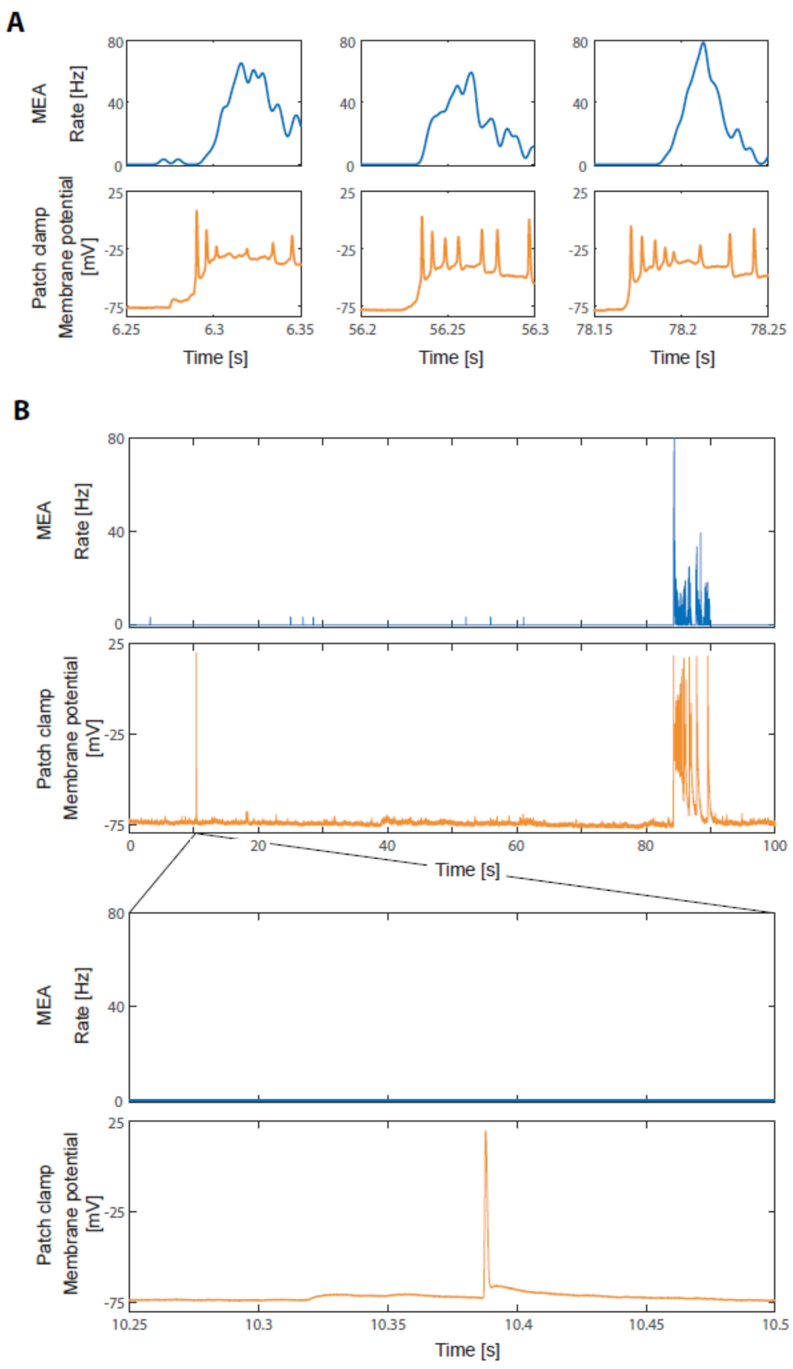

Figure S3. The correlation between the network's activity and the neuronal membrane potential.

A: A zoom-in of Figure 2C in the manuscript. A zoom-in of three of the bursts shown in Figure 2C in the manuscript for the network firing rate, recorded by the extracellular electrodes (top), and the membrane potential of the single neuron recorded by an intracellular electrode (bottom). Results indicate a high correlation between the bursts of the network and the single neuron. A comparison between the timing of the first spike of the patched neuron (or deviation from the baseline membrane potential voltage, e.g. -75 mV) and the initiation of the network bursts measured by the multi-electrode array (e.g. the first moment where the temporal rate exceeds 5 Hz) indicates a time-shift of very few milliseconds in the left and the middle panels, and about 50 milliseconds in the right panel.

B: A single spike recoded by the patch clamp system that does not necessarily reflect a burst of the network. An example where a network burst is accompanied by a burst of the patched neuron (upper two panels). Nevertheless, it is possible to find an isolated evoked spike of the patched neuron with the lack of similar activity in the network (zoom-in of the relevant time-slot is shown in the two lower panels).

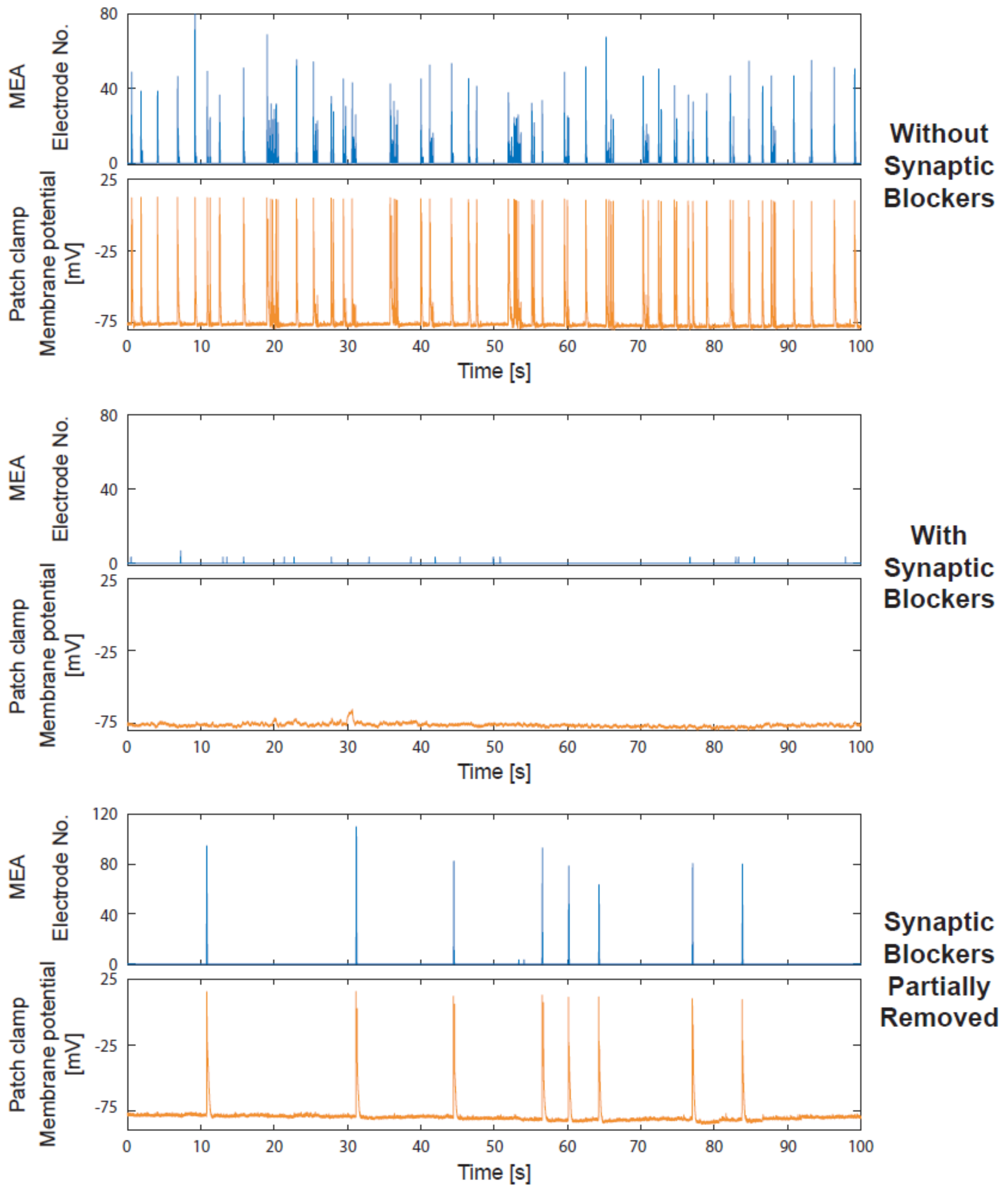

Figure S4. The robustness of the result that a single neuron reflects network activity even under pharmacological manipulations. Upper two panels: The extra-cellular rate activity, recorded simultaneously with the membrane potential of the current-clamped neuron, similar to middle and lower panels of Figure 1B in the manuscript. A high correlation between the timings of the network and the neuronal bursts is evident. Middle panels: When adding synaptic blockers (Online Methods), the cooperative bursts of the network almost vanish as well as the neuronal bursts and postsynaptic potentials. Lower panels: The partial rinse of the blockers revealed short bursts simultaneously both in the patched neuron and in the multi-electrode array.
